# Gender, Resources, and Intimate Partner Violence Against Women in Egypt Before and After the Arab Spring

**DOI:** 10.1177/1077801221992877

**Published:** 2021-03-03

**Authors:** Mariam Abouelenin

**Affiliations:** 1 Lancaster University, UK

**Keywords:** Arab Spring, Egypt, intimate partner violence

## Abstract

This study draws on resource and feminist theories to empirically test the influence of women’s resources and gender performance on psychological and physical intimate partner violence (IPV) in Egypt. Having applied two-stage least squares regressions to nationally representative data from the Demographic and Health Survey (*N =* 11,319), the results show that women’s education and employment reduce their risk of physical IPV and that the effect of women’s employment on IPV is moderated by their spouses’ employment, with the lowest risk of physical IPV observed among employed women with unemployed or blue-collar spouses. Women’s employment and relative education were not associated with the risk of psychological IPV. While education and employment remain among the strongest deterrents of physical IPV, there was no moderation effect found before or after the Arab Spring for psychological and physical IPV.

## Introduction

Recent official statistics on intimate partner violence (IPV) in Egypt have highlighted a significant trend: women are at high risk of exposure to ongoing injurious and life-threatening violence (Duvvury et al., 2015; Ministry of Health and Population [Egypt] et al., 2015), which often takes place within marital relationships. Formally, IPV denotes aggression perpetrated within an intimate relationship that may cause physical, psychological, or sexual harm (World Health Organization, 2012). While physical IPV is more frequently researched, evidence suggests that psychological IPV can be used to maintain power and control over women and may lead to or accompany physical abuse (Follingstad, 2011; Hoff, 2009). Psychological IPV has also been linked to adverse mental and physical health outcomes in women, including depression and suicidal ideation (Jina et al., 2012), and obesity (Yount & Li, 2011). Moreover, estimates of the economic costs of IPV to Egyptian society, including alternative housing, medical, and counseling costs, is US$95 million annually (Duvvury et al., 2015).

Women’s exposure to various forms of male domination has long been normalized in the Arab world (Ammar, 2006; Yount & Li, 2009). Recently, growing political openness and the persistent efforts of female activists have made IPV against women more widely recognized as a serious violation of women’s rights (Ammar, 2006). Most scholarship aimed at identifying the correlates of IPV has relied on two main theoretical frameworks. Resource theory (Blood & Wolfe, 1960) assumes that the spouse with fewer resources, often operationalized as education and income, is more likely to experience IPV (Goode, 1971; Vyas & Watts, 2009). Gender performance theory (West & Zimmerman, 1987), which falls under the rubric of feminist epistemology, is cognizant of gender power dynamics and structural inequalities (Anderson, 1997; Dobash & Dobash, 1979). From this perspective, IPV is considered culturally embedded and encouraged to maintain the patriarchal status quo (Chung et al., 2008; Weitzman, 2014).

Both perspectives have provided tools for understanding the complexities of IPV in Western societies (Anderson, 1997; Goode, 1971). Yet, despite the frequency and severity of IPV in the Arab world (Ammar, 2006; Duvvury et al., 2015), similar scholarship in religious and patriarchal societies remains relatively scarce. Questions about how resources, gender, and power might relate to IPV have not received equal attention. Female education and employment serve as determinants of IPV victimization (Fageeh, 2014; Jewkes, 2002). In many Western societies, women’s rising educational attainment and participation in paid employment have been well-documented (Blossfeld & Kiernan, 2019). Conversely, while many countries in the Arab world, including Egypt, have achieved gender parity in primary- and secondary-level education, women’s employment rates are among the lowest in the world (Assaad & Krafft, 2015). Some studies suggest that cultural norms in the region perpetuate IPV against women and impede women’s social and economic equality (Yount, 2005b; Yount & Li, 2010). Given contrasting views between those who assert that traditional gender roles justify IPV against women and those who argue that women’s resources offer protection, our understanding of the correlates of IPV in patriarchal contexts remains limited.

The aim of this study is twofold. First, I examine the role of women’s education and employment in shaping the incidence of IPV against women in Egypt. Second, I explore these relationships in the context of the events that took place during the Arab Spring. It is not the objective of this study to disentangle whether any changes observed are attributed to social norms, which determine gender roles and the distribution of resources, or to women’s newfound access to the public sphere throughout the Arab Spring. What I aim to do is test how women’s resources shape their exposure to IPV before and after the Arab Spring. I also craft an instrumental variable (IV) two-stage least squares (2SLS) estimation method to explain and disentangle the potential reciprocal relationship between women’s paid employment and IPV (Bhattacharya, 2015; Lenze & Klasen, 2017).

## Theoretical Considerations

### Defining and Measuring IPV

Despite the broad definition of IPV, its operationalization has often been limited to physical IPV (DeKeseredy & Schwartz, 2011) due to hesitancies toward combining physically injurious behavior with psychological abuse under the umbrella term “IPV” (DeKeseredy & Schwartz, 2011). Whereas physical IPV refers to the deliberate use of physical force (Hoff, 2009), psychological IPV refers to a wide range of emotionally abusive behavior, which may involve threats, ridicule, isolation, or public humiliation (Follingstad, 2011). The focus of research on IPV has traditionally been on the former, which is easier to recognize and report (see Appendix A for information on the Egyptian Penal Code). Therefore, to determine how female education and employment relate to different forms of IPV, I distinguish between psychological and physical IPV and examine them side by side.

### Resource Perspectives and IPV Against Women

Several theoretical perspectives inform our understanding of IPV against women. Developed from earlier theories of marital power (Blood & Wolfe, 1960), resource theory argues that resource disparities in marital relations underlie IPV (Goode, 1971; Vyas & Watts, 2009). Women’s resources, both economic and nonpecuniary, determine their bargaining power (Blood & Wolfe, 1960). This power not only signifies the ability to influence family relationships, such as participation in housework and household decision-making (Bargain et al., 2019; Hu, 2019), but may also offer protection from abuse (Bowlus & Seitz, 2006). Thus, advocates of this perspective view spousal resources as the primary organizing principle in marital relations, where the imbalance or balance of resources can motivate the use of IPV.

Marital power reflects and is determined by relational dependence (Goode, 1971; Kalmuss & Straus, 1982). The availability of resources is thought to increase women’s autonomy and offer protection from IPV (Goode, 1971). Many have emphasized the role of women’s education in challenging traditional gender norms (Flake, 2005; Jewkes, 2002), which are often entrenched in sociocultural expectations of female domesticity. Women’s education can improve their access to support networks, making them less likely to rationalize their spouses’ use of IPV (Jewkes, 2002; Yount & Li, 2009) and better equipped to exit abusive relationships (Vyas & Watts, 2009). Similarly, women’s employment arguably strengthens their financial self-reliance, thereby reducing their risk of IPV (Vyas & Watts, 2009). In more gender-egalitarian societies, such as Canada and Peru, researchers have found that women’s employment and education are significant deterrents of IPV (Bowlus & Seitz, 2006; Flake, 2005). Findings from societies dominated by patriarchal ideologies, such as India and Saudi Arabia, do not support this perspective but indicate that cultural expectations that emphasize women’s dependence on their spouses likely curtail their autonomy (Fageeh, 2014; Paul, 2016).

Others suggest that power is relational and depends on spouses’ relative resource contributions (Lundberg & Pollak, 1993; Macmillan & Gartner, 1999). In marriage, power is attained and maintained through a series of exchange processes (Becker, 1991). Women with relatively abundant resources vis-à-vis their spouse can participate in this process by sharing or withholding their resources (Lundberg & Pollak, 1993; Macmillan & Gartner, 1999). The threat of resource deprivation and its impact on men’s bargaining power may deter the use of IPV (Lundberg & Pollak, 1993). Research testing the role of resource bargaining in women’s exposure to IPV has predominantly focused on employment and education. In the United Kingdom, improvements in women’s labor market participation relative to men’s strengthened their bargaining position and were negatively associated with IPV (Anderberg et al., 2016). A U.S. study found that women who were less educated than their spouses were at greater risk of IPV (Kalmuss & Straus, 1982). However, in the absence of gender-egalitarian norms, women’s relative resources are not found to strengthen their bargaining position in India (Weitzman, 2014).

Research on psychological and physical IPV in Egypt assessing the roles of both women’s absolute and relative resources is largely missing. This stems in part from a lack of nationally representative Egyptian data and the absence of questions on the nature and timing of nonphysical forms of abuse in surveys (Ammar, 2006; Yount, 2005b). This has meant that a notable gap in understanding the applicability of resource theories to IPV beyond Western contexts remains.

### Feminist Perspectives and IPV Against Women

One distinction between resource and feminist perspectives lies in their conceptualizations of power. Resource theory emphasizes that power is nonexclusive and varies by the amount of resources an individual possesses. The feminist perspective, however, posits that power is contextually embedded and often reserved for men (Dobash & Dobash, 1979). Patriarchal systems shape social expectations regarding gender roles and power. These roles reinforce men’s superiority by facilitating their disproportionate access to social, economic, and political institutions (Anderson, 1997; Dobash & Dobash, 1979). Similar power inequalities permeate marital relations, increasing women’s vulnerability to men’s control (Yount & Li, 2010).

Gender performance theories posit that women’s and men’s actions are governed by the beliefs of different sociocultural groups (West & Zimmerman, 1987). In societies where patriarchal ideologies hold sway, societal norms define men as primary income-earners and women as homemakers (Mensch et al., 2003). For instance, in most Arab societies, women’s education and labor market participation is discouraged and, in many cases, household income is controlled by the male patriarch (Mensch et al., 2003). The financial organization of patriarchal households (Hu, 2019) can still leave working women financially dependent on their spouses. When women’s gender performance deviates from these norms, their spouses may perceive their masculinity and social status as undermined (West & Zimmerman, 1987).

This perspective illuminates empirical findings that women’s employment and education can challenge dominant norms of male superiority that may legitimize the use of IPV (Chung et al., 2008; Flake, 2005; Weitzman, 2014). Research in India and Peru has shown that wives who are better educated than their spouses are more likely to experience IPV (Flake, 2005; Weitzman, 2014), indicating that violation of traditional gender norms increases the risk of IPV against women. In direct contrast to the predictions of resource theory, among Asian American couples, women whose income was at least equal to that of their partner were at greater risk of IPV (Chung et al., 2008). A study in Mexico found that women’s paid employment increases the risk of IPV when their partner is unemployed and reduces the risk when their partner is employed (Villarreal, 2007).

Women’s gender performance is not evaluated independently from that of their spouses. Rather, it is interpreted relationally and with reference to context. Gender performance theories suggest that women whose education and employment status conflict with societal norms are at greater risk of IPV (Chung et al., 2008; Flake, 2005; Weitzman, 2014), particularly in societies like Egypt, where rigid gender roles prevail (Yount, 2005b; Yount & Li, 2010). Conversely, spouses who encounter threats to their masculinity when outperformed by women may also use IPV to re-establish their power.

### Social and Economic Changes in Egypt Before and After the Arab Spring

Mass protests throughout the Arab world began in 2010 and have since become widely known as the Arab Spring. In 2011, these protests spread to Egypt and have resulted in significant social and economic changes. Traditionally, gender norms restrict women’s roles to the private sphere of the family (Mensch et al., 2003). During the protests, however, women across the country were granted greater access to the public sphere and some even became politically active (Bargain et al., 2019). The adverse economic consequences of the Arab Spring, such as rising male unemployment and poverty rates (Abdou & Zaazou, 2013; Assaad & Krafft, 2015) and higher food and fuel prices (Abdou & Zaazou, 2013), have also increased women’s involvement in public life. Specifically, changes to women’s economic participation are observed as they take on roles that were previously reserved for men, such as paid employment, out of economic necessity (El-Mallakh et al., 2018). At the same time, IPV remains prevalent and has risen in recent years (Duvvury et al., 2015). The estimated prevalence of IPV among women in the Arab world ranges from 8–65% (Boy & Kulczycki, 2008). By comparison, the prevalence of physical and psychological violence in Egypt among ever-married women is reported to be 25 and 19%, respectively (Ministry of Health and Population [Egypt] et al., 2015).

Women’s educational attainment has improved considerably in recent years. Egypt has been moving toward gender parity in primary and secondary school enrolment (Elbadawy, 2015). Improvements have also been observed in women’s education relative to their spouses. According to the Central Agency for Public Mobilization and Statistics (2018), 9.8% of wives and 6.9% of spouses were illiterate in 2016, compared with 17.7 and 10.9% in 2007, respectively. However, despite increased female education, women still lag behind men in labor force participation. Women’s employment rates ranged from 18–23% between 2002 and 2014 (World Bank, World Development Indicators, 2019a), while men’s employment rates over the same period ranged between 68 and 75% (World Bank, World Development Indicators, 2019b). The differences in men’s and women’s employment can be partly attributed to cultural expectations that inhibit women’s economic participation.

The assumption that education reduces IPV in the West is predicated on the idea that education enables women to question and challenge discriminatory gender norms (Jewkes, 2002). This is underpinned by the development of women’s aspirations that transcend their domestic roles (Jewkes, 2002). This premise, however, may not hold in Egypt as the value of women’s education is based on its importance for family functionality. Rather than fulfilling women’s intellectual endeavors and enhancing their labor market outcomes, education is widely deemed to facilitate women’s domestic responsibilities by ensuring they raise well-educated children who will uphold patriarchal religious beliefs (Al-Sharmani, 2010; Mensch et al., 2003). This means that women’s education and its use in Egypt constitute a resource of cultural preservation rather than empowerment.

Women’s gender roles in the Egyptian family are changing, repositioning them as income-earners due to deteriorating economic conditions. In 2012, 20% of men were irregular wage workers, up from 9% in 2006 (Assaad & Krafft, 2015). Men’s deteriorated labor market outcomes have also been accompanied by a surge in the prices of consumer goods (Abdou & Zaazou, 2013), increasing the financial strain on Egyptian families. The decline of men’s employment and economic security has entailed a relative increase in women’s labor force participation rates, as reflected in the narrowing gender gap and an increase in women’s weekly hours of paid work (El-Mallakh et al., 2018). As Egyptian women’s participation in stereotypically masculine domains, such as paid employment, has been justified on the grounds of fulfilling the family’s financial needs (El-Mallakh et al., 2018), the above trends suggest that in unfavorable economic conditions, women’s employment may be considered more socially permissible since the Arab Spring.

Women and men may waive traditional gender expectations in favor of the family’s economic subsistence. The potential conflict between the economic necessity of women’s employment and traditional beliefs that define women as inferior to men, raises questions about how new gender arrangements may impact IPV against women since the Arab Spring. Arguably, the pressure of economic crises may necessitate women’s paid employment to help support the family, which can lead to more gender-equitable norms and less IPV.

### Hypotheses

The discussion above can be summarized with the following four hypotheses. Hypothesis 1 focuses on women’s absolute resources and tests the assumptions of individual autonomy theory, Hypotheses 2 and 3 focus on women’s relative resources and tests the assumptions of resource bargaining and gender performance theory, and Hypothesis 4 compares the effects of women’s education and employment before and after the Arab Spring:

**Hypothesis 1 (H1):** Educated women are less likely to experience IPV than their less educated counterparts (H1A); employed women are less likely to experience IPV than their unemployed counterparts (H1B).
**Hypothesis 2 (H2):** Women who are better educated than their spouses are less likely to experience IPV than women who are less or similarly educated than their spouses (H2A); employed women are less likely to experience IPV when their male spouses are unemployed or in low-status occupations compared with other men (H2B).
**Hypothesis 3 (H3):** Women who are better educated than their spouses are more likely to experience IPV than women who are less or similarly educated compared with their spouses (H3A); employed women are more likely to experience IPV when their male partners are unemployed or in low-status occupations compared with other men (H3B).
**Hypothesis 4 (H4):** The positive association between women who are better educated than their spouses and IPV was stronger before, compared with after, the Arab Spring (H4A); the positive association between women’s employment and IPV was stronger before, compared with after, the Arab Spring (H4B).

## Method

### Data and Sample

The analysis is based on the Demographic and Health Survey (DHS), one of the most comprehensive repositories of data on IPV against women in Egypt, first conducted in 1988. The survey data were collected using multistage sampling. It has since been repeated in seven waves (standard DHS), providing the largest nationally representative sample of Egyptian women. All ever-married women aged 15–49 who were present in the household the night before the survey were eligible for the in-person interview (Ministry of Health and Population [Egypt] et al., 2015). In the 2005 and 2014 waves, a special domestic violence (DV) module was administered to a subsample of women in one third of the households and inquired about women’s exposure to physical and psychological IPV. One eligible woman was randomly selected from each household to complete the DV interview (Ministry of Health and Population [Egypt] et al., 2015).

The analytical sample was restricted to the 2005 and 2014 DHS waves when the DV module was available. This also provides a suitable timeframe that covers the periods prior to and after the 2011 Arab Spring. I then excluded all women who did not participate in the DV module (*N* = 28,930). Of the remaining 12,306 women, those who were widowed (*N* = 436), divorced (*N* = 256), or separated (*N* = 71) were excluded due to the unavailability of spousal information. The low divorce rate reflects the universality of marriage in Egypt, where the national divorce rate was approximately 2–3% in 2015 (Salem, 2015). A total of 224 women were omitted as a result of item nonresponse on the variables used in the analysis, such as spouse’s occupation, consanguinity, religion, and marriage order. Little’s test of missing completely at random (MCAR) confirmed the listwise deletion of missing cases was MCAR (Li, 2013). The final analytical sample included 11,319 currently married women aged 15–49 years with complete data on all variables. Sampling weights were used in all analyses to account for the sample design.

### Estimation Strategy

To test resource and gender performance theories, I used women’s education and employment to predict IPV. However, the literature suggests a bidirectional relationship between employment and IPV (Bhattacharya, 2015; Lenze & Klasen, 2017): women’s employment may determine their risk of IPV and IPV may in turn impinge on women’s decisions to enter or exit the labor market. From an exposure reduction standpoint, scholars argue that abused women might intentionally pursue employment to reduce the amount of time spent in the vicinity of their abuser (Bhattacharya, 2015). Others have suggested a relationship in the opposite direction, whereby IPV leads to employee absenteeism due to physical and mental health issues (Riger & Staggs, 2004). The potential bidirectionality and omitted variable bias means that an ordinary least squares (OLS) regression would provide inconsistent parameter estimates.

To account for the potential bidirectional relationship between women’s employment and IPV, I use an IV estimator. One form of this estimator is 2SLS. In the first stage, I regressed the endogenous variable (i.e., women’s employment) on the IVs and covariates. In the second stage, I regressed IPV on the predicted probability of women’s employment obtained from the first-stage regression (see Appendix B). All models were estimated using the *ivreg2* package in Stata, where the two equations are estimated jointly to obtain corrected standard errors (Baum et al., 2007). An instrument’s validity depends on three fundamental assumptions (Kitagawa, 2015). First, it is correlated with the endogenous variable (i.e., employment). Second, it does not directly impact women’s likelihood of experiencing IPV (i.e., it is not correlated with the error terms of the outcome variable). Third, its effect on IPV is unconfounded (i.e., it must be strongly associated with employment but not associated with our covariates). To test the relevance of the IVs, the Anderson canonical correlation likelihood ratio test was used. To determine whether the IVs were correlated with the error term, the Hansen–Sargan over-identification test was used. Finally, to evaluate the overall strength of the instruments, I used the Cragg–Donald test for weak instruments.

Separate models were fitted for women’s exposure to psychological (Model 1) and physical violence (Model 2). I ran three models: (a) key predictors and covariates, (b) added an interaction between women’s and spouses’ employment, and (c) added interactions between survey year, women’s employment, and women’s relative education. I also tested a three-way interaction between women’s employment, spouses’ employment, and survey year. The interaction was not statistically significant, it did not affect the results for the other variables, and its inclusion did not improve the model fit. Therefore, I excluded it from the analyses reported in this article. To interpret the interaction effects, I calculated the predictive margins of the interactions and used the *lincom* function to compare slopes. The variance inflation factor (VIF) test for multiple collinearity yielded VIF values below the threshold of 2.5 for all variables, except for the number of children.

### IVs

To address the potential endogeneity of women’s employment, I used two IVs selected based on prior theories and empirical tests. The first instrument was a continuous measure for the number of usual residents who had slept in the house the previous night and were listed in the household schedule (see Appendix C). This measure was top-coded at the 99th percentile to reduce the influence of outliers. The number of household members should be strongly correlated with women’s employment. If resident members are of state pension age and can contribute to household income needs, women may withdraw from the labor force (Maurer-Fazio et al., 2011). In Egypt, social norms oblige adult children to care for aging family members so that the overwhelming burden of unpaid elderly care is typically handled by women, which may curtail their employment opportunities. It is also plausible that the number of household members has no direct effect on women’s exposure to IPV. The patrilocal extended family has long been the ideal family structure in Egypt (Yount, 2005a), which is likely to limit married women’s access to natal kin who can impact their risk of IPV. Marital relations also operate following the patrilineal tradition whereby men assume the role of household head and are entitled to power over their spouses through various means, including IPV (Ammar, 2006). This in turn may discourage family intervention in marital conflict.

The second instrument captures geographical differences in female employment rates, using a continuous measure capturing the governorate average of women’s employment. Egypt’s DHS (2005, 2014) is grouped into geographical units known as governorates, indicating the region in which the female respondent was interviewed (see Appendix D). The governorate average of women’s employment should be related to the probability of women’s own employment but should not be correlated with IPV, except through its effect on women’s labor market participation.

### Outcome Variables

The 2005 and 2014 DHS contained a DV module that was adapted using a Revised Conflict Tactics Scale (CTS2; Straus et al., 1996), which contains a rich array of information on physical and psychological IPV. Women who reported having experienced either form of violence were asked about the frequency of violence over the 12 months prior to the survey. As interpretations of what constitutes violence may vary between women, the inclusion of additional items and the use of behavior-specific questions in the CTS2 enhances content validity and better captures IPV against women (Straus et al., 1996).

To measure physical IPV, the women were asked whether their spouses had, in the preceding 12 months, (a) pushed, shaken, or thrown something at them; (b) slapped them; (c) punched or hit them with an object; (d) attempted to strangle or burn them; or (e) kicked or dragged them, respectively. To measure psychological IPV, the women were asked whether their spouses had, in the preceding 12 months, (a) humiliated them or (b) threatened them with harm. The responses were recorded on a 3-point scale indicating the frequency of violence: “not at all” (0), “sometimes” (1), and “often” (2). Using principal component analysis and varimax rotation, the five physical IPV items (eigenvalue = 2.88, Cronbach’s α = .80) and two psychological IPV items (eigenvalue = 1.49, Cronbach’s α = .62) were loaded on two distinct components. The Bartlett method was then used to extract two composite scores, whereby higher scores indicated higher prevalence of psychological or physical IPV in the preceding 12 months.

### Key Predictors

#### Spouses’ education

The women were asked about their own and their spouses’ respective level of education. The response categories include “no education” (25%), “primary” (13%), “secondary” (51%), and “higher-level” (11%). Using spousal information provided by the female respondents, I constructed four variables to measure spouses’ relative education. The first three are dummy variables, distinguishing couples in which “the wife is better educated” (14%), “the spouse is better educated” (26%), and in which “both spouses are educated to the same level” (60%), respectively. The fourth was constructed following Schwartz and Han (2014) and is a continuous variable measuring the absolute difference in the level of education between spouses. The variable ranges from 1–3 for spouses with different educational levels, whereas educationally homogamous couples were coded as 1.

#### Women’s employment

I included a dummy variable distinguishing unemployed (84%) and employed (16%) women, which was based on whether the female respondent worked outside the home in the preceding 12 months and was paid in cash, cash and in-kind, or in-kind only payments.

#### Spouse’s employment

I included a dummy variable that takes on the value of 0 if the spouse is a “white-collar worker” (30%) and 1 if the spouse is a” blue-collar worker or unemployed” (70%). I combined blue-collar and unemployed spouses into one category as distinguishing between them does not yield statistically significant results (*p* > .10).

### Covariates

I also controlled for several characteristics that are likely to influence IPV against women in Egypt. Research has found that IPV is more common among women who have children (Yount, 2005b). Therefore, I included a dummy variable capturing whether the female respondent had at least one child (81%) or no children (19%). As consanguineous marriage may increase women’s access to natal kin and mitigate the risk of IPV (Yount & Li, 2010), I distinguished between women married to nonrelatives (63%), first or second paternal cousins (18%), first or second maternal cousins (9%), or other relatives (10%). To account for geographical differences, I included a categorical variable distinguishing women residing in urban governorates (16%), Lower Egypt (36%), Upper Egypt (43%), and Frontier governorates (5%).

Given that age differences between spouses can impact women’s ability to behave independently and may increase their risk of IPV victimization (Vyas & Jansen, 2018), I controlled for the respondents’ ages and the age gaps between spouses. After subtracting the spouse’s age from the respondent’s age, the age gap variable was grouped into five categories: (a) wife older than spouse by 5 years or less ([−5, −1]) (3%), (b) no age gap between spouses ([0]) (4%), (c) wife younger than spouse by 1–5 years ([1, 5]) (34%), (d) wife younger than spouse by 6–12 years ([6, 12]) (48%), and (e) wife younger than spouse by 13–25 years ([13, 25]) (11%). As higher-order marriages are more likely to be polygamous and polygamy is associated with a higher risk of IPV (Vyas & Jansen, 2018), I distinguished between women who married once (98%) and more than once (2%). I also controlled for women’s age at first marriage, rural/urban residence, religion (Muslim vs. Christian), whether they had experienced physical abuse by their mother or father since age 15, and a household wealth index divided into quintiles from the poorest to the richest. Descriptive statistics for all variables used in the analyses are presented in Table 1.

**Table 1.** Sample Characteristics Married Women Aged 15–49 years (*N* = 11,319).

Key variables	Min	Max	Mean/%	*SD*
Intimate partner violence (IPV)				
Psychological	−0.31	9.04	0	1.22
Physical	−0.51	16.20	0	1.69
Women’s relative education				
Husband better educated than wife (H > W)	0	1	26.34	
Husband and wife similarly educated (H = W)	0	1	59.20	
Wife better educated than husband (W > H)	0	1	14.50	
Women’s absolute education				
No education	0	1	24.88	
Primary	0	1	13.40	
Secondary	0	1	50.55	
Higher education	0	1	11.19	
Educational gap between spouses	1	3	1.12	0.39
Women’s employment (ref: unemployed)	0	1	15.80	
Spouse white-collar worker (ref: blue-collar or unemployed)	0	1	30.50	
2014 survey year (ref: 2005)	0	1	52.50	
*Covariates*				
Family of origin violence (ref: no violence)	0	1	19.10	
Husband/other males present during IPV questions (ref: not present)	0	1	3.24	
Age gap^a^				
–5/–1	0	1	2.82	
0	0	1	3.54	
1/5	0	1	34.50	
6/12	0	1	48.30	
13/25	0	1	11.10	
Age	15	49	29.33	9.49
Married more than once (ref: married only once)	0	1	2.29	
Age at first marriage	12	32	19.11	3.91
Relational status of husband				
Nonrelative	0	1	62.80	
First or second paternal cousin	0	1	17.74	
First or second maternal cousin	0	1	9.37	
Other relative by blood or marriage	0	1	10.11	
Christian (ref: Muslim)	0	1	3.96	
Urban (ref: rural)	0	1	39.60	
Governorate				
Urban governorates	0	1	15.90	
Lower Egypt	0	1	35.63	
Upper Egypt	0	1	43.30	
Frontier governorates	0	1	5.23	
Has at least one child (ref: no children)	0	1	81.03	
Wealth quintiles				
Poorest	0	1	18.60	
Poorer	0	1	20.50	
Middle	0	1	20.41	
Richer	0	1	20.50	
Richest	0	1	20.12	
Total number of household members^b^	2	14	5.17	2.32
Governorate^c^ average of women’s employment	0.06	0.49	0.18	0.07

*Note.* Min = minimum value. Max = maximum value. SD = standard deviation. For dummy variables, 0 = No and 1 = Yes. Mean values reported for continuous variables and percentages for dummy and categorical variables. Percentages may not add up to 1 due to rounding. ^a^ Bottom- and ^b^ top-coded at the 1st and 99th percentiles. ^c^ Based on 27 governorates. Weighted statistics with unweighted sample size.

## Results

### Descriptive Statistics

**Figure 1.**
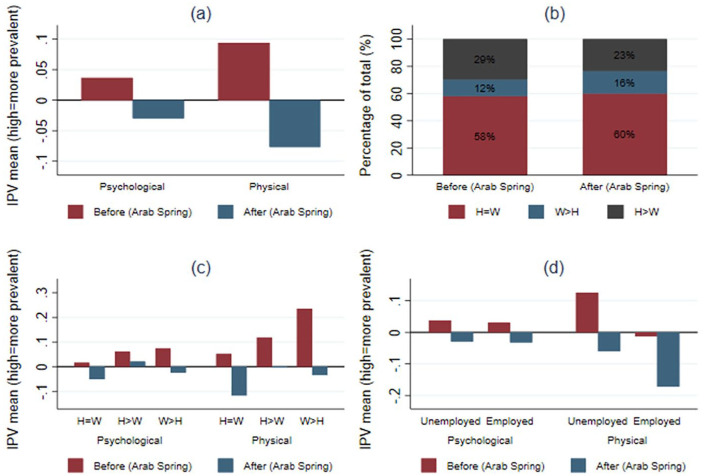
Descriptive statistics of women’s employment, relative education, and intimate partner violence before and after the Arab Spring. *Note. N* = 11,319 women. Unweighted statistics with unweighted sample size. Panel A: psychological and physical IPV before and after the Arab Spring. Panel B: women’s relative education before and after the Arab Spring. Panel C: physical and psychological IPV by women’s relative education before and after the Arab Spring. Panel D: physical and psychological IPV by women’s employment before and after the Arab Spring. IPV = intimate partner violence. H = W = both spouses educated to the same level. W > H = wife is better educated. H > W = husband is better educated. Before the Arab Spring = 2005 wave. After the Arab Spring = 2014 wave.

Panel A of Figure 1 presents the descriptive statistics for psychological and physical IPV before and after the Arab Spring. As higher scores indicate a higher level of IPV, the results show a decrease in the prevalence of psychological and physical IPV after the Arab Spring. This decline is more pronounced for physical than psychological IPV.

Panel B of Figure 1 shows the distribution of spouses’ education before and after the Arab Spring. More women were less educated than their spouses before the Arab Spring than after (29 vs. 23%), while the percentage of women who were better educated than their spouses was higher after the Arab Spring period (12 vs. 16%). These results concur with previous findings that gender disparities in university attendance have narrowed substantially in Egypt over the last decade (Elbadawy, 2015).

In Panel C of Figure 1, the results also show significant differences in the prevalence of physical and psychological IPV according to women’s relative education vis-à-vis that of their spouses. Before the Arab Spring, the prevalence of physical and psychological IPV was higher among women who were better educated than their spouses than women who were equally or less educated than their spouses. After the Arab Spring, the prevalence of physical and psychological IPV was higher among women who were less educated than their spouses than women who were equally or better educated than their spouses. As Panel D of Figure 1 indicates, the prevalence of IPV also varies by women’s employment status and over time. The prevalence of psychological and physical IPV for employed women has significantly decreased since the Arab Spring.

### 2SLS Results

Table 2 presents the results of the 2SLS regression models predicting psychological IPV against women in the left panel and physical IPV in the right panel. Three tests were conducted to assess the strength and validity of the IVs used (Hu, 2019; Shen et al., 2016 ). Both instruments, the number of household members and the governorate average of women’s employment, were strongly correlated with women’s employment in the first-stage regression model (*p* < .01 and *p* < .001, respectively). To assess the instruments’ relevance, the Anderson canonical correlation statistic for under-identification confirmed that the equation was identified (χ^2^ = 460.94, *p* < .001). The Sargan–Hansen statistic was small and statistically insignificant (*p* > .10), confirming that the IVs were uncorrelated with the error term. Finally, the results of the Cragg–Donald Wald *F* statistic (*F* = 234.54) exceeded the Stock–Yogo weak instruments test rule of thumb of 10 (Stock & Yogo, 2005).

**Table 2.** Two-Stage Least Squares Regression Models Predicting Psychological (Panel A) and Physical (Panel B) IPV in the Previous 12 Months (*N* = 11,319).

	Psychological			Physical		
	Model 1A	Model 1B	Model 1C	Model 2A	Model 2B	Model 2C
	*B* (SE)	*B* (SE)	*B* (SE)	*B* (SE)	*B* (SE)	*B* (SE)
Women’s employment (ref: unemployed)	−.08 (.27)			−.80* (.34)		
Employed × Blue-collar and unemployed		−.01 (.31)			−.93** (.32)	
Employed × White-collar		−.25 (.41)			−.16 (.67)	
Employed × 2005			−.15 (.26)			−.99** (.33)
Employed × 2014			−.03 (.72)			−.25 (.76)
White-collar worker (ref: blue-collar and unemployed)	−.05 (.04)	−.01 (.09)	−.05 (.04)	−.02 (.05)	−.14 (.11)	−.02 (.05)
Relative education (ref: H = W)						
W > H	.05 (.05)	.04 (.05)		.09 (.06)	.12† (.07)	
H > W	−.01 (.05)	−.01 (.05)		−.10 (.07)	−.11† (.06)	
H > W × 2005			−.03 (.06)			−.20* (.08)
H > W × 2014			.02 (.07)			−.01 (.08)
W > H × 2005			.11 (.08)			.21† (.11)
W > H × 2014			.01 (.07)			.03 (.06)
Educational distance	.07 (.07)	.07 (.07)	.07 (.07)	.03 (.06)	.03 (.06)	.03 (.06)
Women’s education (ref: none)						
Primary	.04 (.09)	.04 (.09)	.03 (.09)	−.13 (.10)	−.13 (.10)	−.14 (.10)
Secondary	−.14* (.06)	−.13* (.05)	−.14* (.06)	−.38*** (.10)	−.41*** (.09)	−.39*** (.10)
Higher	−.19† (.11)	−.15 (.10)	−.19 (.15)	−.46*** (.13)	−.60*** (.18)	−.53** (.16)
Physically hurt by father or mother (ref: no)	.21*** (.06)	.21*** (.05)	.21*** (.05)	.57*** (.08)	.57*** (.08)	.58*** (.08)
Husband/other male present for IPV questions	.02 (.08)	.02 (.08)	.02 (.08)	.03 (.12)	.02 (.13)	.03 (.13)
Wealth quintiles (ref: poorest)						
Poor	−.04 (.06)	−.04 (.06)	−.04 (.06)	−.11 (.08)	−.12 (.08)	−.10 (.08)
Middle	−.01 (.07)	−.00 (.07)	−.00 (.07)	−.12 (.08)	−.13 (.08)	−.10 (.08)
Rich	−.10 (.06)	−.10 (.06)	−.10 (.07)	−.09 (.08)	−.10 (.09)	−.08 (.09)
Richest	−.17* (.07)	−.17* (.07)	−.17* (.07)	−.20* (.09)	−.21* (.09)	−.18† (.10)
Married more than once (ref: married only once)	.18† (.10)	.18† (.10)	.18† (.10)	.51*** (.15)	.51*** (.15)	.52*** (.16)
Age at first marriage	−.01 (.01)	−.01 (.01)	−.01 (.01)	.01 (.01)	.00 (.01)	.00 (.01)
Consanguinity (ref: husband nonrelative)						
First or second paternal cousin	−.08 (.05)	−.08 (.05)	−.08 (.05)	−.18** (.05)	−.18** (.06)	−.18** (.06)
First or second maternal cousin	−.05 (.05)	−.05 (.05)	−.04 (.05)	−.09 (.06)	−.09 (.07)	−.08 (.06)
Other relative by blood or marriage	−.08† (.04)	−.08† (.04)	−.08† (.05)	.00 (.08)	−.00 (.08)	−.01 (.07)
Age	−.01† (.00)	−.01† (.00)	−.01 (.00)	−.01** (.00)	−.01** (.00)	−.01* (.00)
Age gap (ref: −3, −1)						
0	−.13 (.10)	−.13 (.10)	−.14 (.11)	−.02 (.14)	-.04 (.14)	−.05 (.14)
1, 5	−.22* (.09)	−.22* (.09)	−.23* (.09)	−.16 (.11)	−.18 (.11)	−.17 (.10)
6, 12	−.27** (.09)	−.27** (.09)	−.28** (.09)	−.26* (.11)	−.28* (.11)	−.27* (.11)
13, 25	−.18† (.10)	−.18† (.10)	−.19† (.10)	−.27* (.13)	−.29* (.13)	−.28* (.12)
Christian (ref: Muslim)	−.02 (.05)	−.02 (.06)	−.02 (.06)	−.07 (.07)	−.08 (.08)	−.07 (.07)
Urban (ref: rural)	.17** (.05)	.17** (.06)	.16** (.06)	.13* (.06)	.14* (.06)	.13* (.06)
Region (ref: urban governorates)						
Lower Egypt	.00 (.05)	.01 (.05)	.01 (.06)	−.01 (.07)	−.03 (.07)	−.02 (.07)
Upper Egypt	−.02 (.05)	−.01 (.05)	−.02 (.06)	−.03 (.06)	−.06 (.06)	−.04 (.06)
Frontier governorates	−.08 (.07)	−.07 (.08)	−.07 (.07)	−.22*** (.07)	−.25*** (.07)	−.23*** (.07)
Has at least one child (ref: no children)	.24*** (.06)	.24*** (.06)	.24*** (.06)	.29*** (.08)	.29*** (.08)	.29*** (.08)
2014 (ref: 2005)	.02 (.04)	.01 (.04)	−.00 (.11)	−.09 (.06)	−.08 (.07)	−.22 (.12)
Intercept	.39* (.19)	.34 (.19)	.42* (.21)	.60* (.23)	.77** (.27)	.72** (.26)
Anderson canonical correlation LR test (χ^2^)	460.94***	211.02***	139.74***	460.94***	211.02***	139.74***
Cragg–Donald *F* statistic	234.54***	53.08***	35.03***	234.54***	53.08***	35.03***
Hansen–Sargan (χ^2^)	.677	.802	3.311	.253	2.842	6.177

*Note.* SE = standard error. LR = likelihood ratio. IPV = Intimate partner violence. H = W = both spouses educated to the same level. W > H = wife is better educated. H > W = husband is better educated. Reference categories and standard errors are in parentheses. Weighted statistics with unweighted sample size.

† *p* < .10 *p < .05; **p < .01; ***p < .001 (two-tailed tests).

#### Psychological IPV

Model 1A analyses the effects of women’s absolute and relative education, along with the covariates. In line with individual autonomy theory, Hypothesis 1A proposed that educated women are less likely to experience IPV than their less educated counterparts. This hypothesis was supported: compared with women with no education, women who received secondary education were significantly less likely to experience psychological IPV (β = −.14, *p* < .05). Women’s higher education was also marginally negatively associated with psychological IPV (β = −.19, *p* < .10). These results suggest that women’s education enhances their autonomy and reduces IPV. Women’s relative education was not statistically significant, lending no support to bargaining theory (Hypothesis 2A) or gender performance theory (Hypothesis 3A).

Hypothesis 1B, derived from individual autonomy theory, predicts that employed women are less likely than their unemployed counterparts to experience IPV. The results do not support this hypothesis. While the coefficient for women’s employment is negative, it was not statistically significant at the 10% level in Model 1A. To test the moderating role of the spouse’s employment on the relationship between women’s employment and psychological IPV, I include an interaction term between women’s and their spouses’ employment in Model 1B. The interaction term was not statistically significant at the 10% level, meaning that neither bargaining theory (Hypothesis 2B) nor gender performance theory (Hypothesis 3B) is supported.

#### Physical IPV

Model 2A predicts women’s exposure to physical IPV. As in the case of psychological IPV, the results support individual autonomy theory Hypothesis 1A: better educated women are less likely than their less educated counterparts to experience physical IPV. The results indicate that compared with women with no education, women who received secondary and higher education were significantly less likely to experience physical IPV (β = −.38, *p* < .001 and β = −.46, *p* < .001, respectively). The results do not support bargaining theory (Hypothesis 2A) or gender performance theory (Hypothesis 3A). Although the direction of the coefficients for women’s relative education align with gender performance theory, they were not statistically significant at the 10% level.

Model 2A also tested the association between women’s employment and physical IPV. The results support Hypothesis 1B derived from individual autonomy theory, which predicts that employed women are less likely than unemployed women to experience IPV. Specifically, women’s employment was significantly and negatively associated with physical IPV (β = −.80, *p* < .05). I introduced an interaction term between women’s and their spouses’ employment in Model 2B. Hypothesis 2B, which states that employed women are less likely to experience IPV when their male partners are unemployed or in low-status occupations, was supported by the results. The negative association between women’s employment and physical IPV was stronger for women with blue-collar or unemployed spouses than women with white-collar spouses (β = −.93, *p* < .01 and β = −.16, *ns*, respectively). The between-coefficient difference was also statistically significant at the 10% level.

#### Differences before and after the Arab Spring

Hypothesis 4A predicted that the positive association between women who are better educated than their spouses and IPV would be stronger before, compared with after, the Arab Spring. This was not supported by the results for psychological IPV in Model 1C: the interaction term between women’s relative education and survey year was not statistically significant at the 10% level. Hypothesis 4B, which predicts that the positive association between women’s employment and IPV would be stronger before, compared with after, the Arab Spring, was also not supported by the results in Model 1C. The interaction between women’s employment and survey year was not statistically significant at the 10% level.

Regarding physical IPV, the results from Model 2C do not support Hypothesis 4A, as the between-coefficient difference was not statistically significant at the 10% level. Similarly, I find no support for Hypothesis 4B in terms of the association between women’s employment and physical IPV before, compared with after, the Arab Spring. The between-slope and between-coefficient differences were not statistically significant at the 10% level.

### Sensitivity Analysis

I conducted three sets of sensitivity analyses to evaluate the robustness of the findings. First, I repeated the analyses using the alternative measures of lifetime prevalence of psychological and physical IPV. The direction and size of the coefficients were consistent with those for the 2SLS models across the different model specifications. Second, to assess the robustness of model outcomes to the inclusion of additional covariates, I added a categorical variable for marriage duration. The coefficients were statistically insignificant at the 10% level and the results remained unchanged. Third, using binomial logistic regressions, I repeated the analyses based on a binary measure for exposure to physical and psychological IPV in the preceding 12 months. For this, the binary outcome variable distinguished women who experienced none or any physical and psychological IPV, respectively, in the preceding 12 months. The results were also consistent with those for the 2SLS models.

## Discussion

Despite the high prevalence of IPV in the Arab world, empirical evidence on the correlates of IPV in these societies remains limited. Building on existing research conducted in Egypt (Yount, 2005b; Yount & Li, 2009, 2010), this study makes several new contributions by (a) examining the correlates of different forms of IPV; (b) expanding the analysis to include women’s employment and harnessing a 2SLS regression design to account for the bidirectional relationship between employment and IPV; and (c) comparing the relationships between women’s resources, gender performance, and IPV before and after the Arab Spring.

Consistent with individual autonomy theory (Goode, 1971), the results show that women’s education is negatively associated with risk of physical IPV. This is observed for women who receive secondary or higher-level education. Educated women are more likely to support and pursue more equitable gender roles. Most women in Egypt complete their education before marriage, potentially allowing them to apply their education in the spouse selection process. IPV risk may decline if women select spouses who share similar values, which discourage the use of violence. As autonomy theory suggests, education reduces women’s dependence on their spouse and empowers them to leave abusive relationships. However, the West’s social acceptance of divorce is not shared by the Arab world. The centrality of marriage and family in Egyptian culture persists, and divorce continues to be stigmatized. Hence, the effectiveness of education in reducing IPV against women in Egypt may be achieved by mitigating the risks of IPV via spouse selection or by resisting IPV within marriage. The insignificant effect of women’s relative education contradicts both bargaining and gender performance theories. Specifically, the results suggest that, at least when controlling for women’s absolute education, women’s relative education is not associated with psychological or physical IPV.

Although some studies have applied IV approaches to explain the endogeneity of women’s employment (Bhattacharya, 2015; Lenze & Klasen, 2017), none have applied such methods to IPV in Egypt. This study is the first to have explicitly controlled for the endogeneity of women’s employment in predicting IPV in Egypt. No evidence of a relationship between women’s employment and psychological IPV exists; however, the results show a negative relationship between women’s employment and physical IPV. While this finding provides support for individual autonomy theory (Goode, 1971), disentangling whether it is the economic empowerment or necessity of employment that offers protection against IPV lies beyond the scope of this study. Nevertheless, the results suggest that women’s economic resources and their potential to threaten their spouses’ ability to meet gender role expectations does not translate into a higher risk of physical IPV. Instead, women’s involvement in paid work can deter certain forms of IPV.

I further analyzed the effect of women’s employment vis-à-vis their spouses’ employment on IPV. The spouses’ employment moderates the relationship between women’s employment and their risk of IPV. Most notably, the negative association between women’s employment and physical IPV is stronger among women with unemployed or blue-collar spouses. This finding contrasts with gender performance theories (West & Zimmerman, 1987), which we might expect to hold in contexts where gender roles are shaped by patriarchal norms. Instead, the evidence supports the idea that women’s employment affords them greater bargaining power when their spouses are in low-status occupations. Alternatively, while Egyptian society might disapprove of women’s atypical gender performance, the financial maintenance of the family may override the spouse’s discontent and use of IPV. Although this finding does not necessarily invalidate the importance of cultural norms in Egypt, it suggests that the results of studies conducted in Western settings are partially generalizable.

The findings also suggest that the predictions of individual autonomy theory retain their pre-Arab Spring relevance. Whereas previous studies have explored women’s decision-making autonomy and employment as proxies for women’s empowerment after the Arab Spring (Bargain et al., 2019; El-Mallakh et al., 2018), I focused on their risk of IPV. Based on the widespread assumption that women’s political participation and improved labor market outcomes during and after the revolution would bring about more equitable gender roles (Bargain et al., 2019; El-Mallakh et al., 2018), I hypothesized that I would find less support for gender performance theories after the Arab Spring. Instead, women’s education and employment after the Arab Spring did not provide more effective protection against IPV compared with before the Arab Spring. The results indicate that these short-lived revolutions have failed to provide women with the leverage to challenge traditional gender roles and reduce their risk of IPV in Egypt. To the extent that the Arab Spring-related changes altered the relationship between women’s resources and IPV, the findings suggest that political transitions are limited in their ability to empower women.

Finally, the results underscore the importance of analyzing and comparing the correlates of both physical and psychological IPV. The associations identified in the 2SLS regression analyses show a different pattern of relationships between women’s resources and different forms of IPV. Women’s education and employment emerged as deterrents of physical IPV. Goode (1971) suggested that the availability of resources increases women’s individual autonomy and can offer protection from IPV. This view receives consistent support in the models that predict physical IPV. In accordance with bargaining theory (Lundberg & Pollak, 1993; Macmillan & Gartner, 1999), women’s employment vis-à-vis their spouses’ is also negatively associated with their risk of physical IPV when their spouses are unemployed or in low-status occupations compared with other men. I also found that women’s education was only marginally negatively associated with psychological IPV and found no association between women’s employment and psychological IPV. Women’s relative education and employment vis-à-vis their spouses’ was also not associated with psychological IPV. Therefore, women’s education, employment, and employment vis-à-vis their spouses’ can safeguard against physical IPV. However, they do not appear to predict the risk of psychological IPV; that is, neither resource theories nor feminist theories receive strong support. These results are sustained when the moderating effect of the Arab Spring is considered.

This study has several limitations. First, the analysis was based on cross-sectional data on IPV, which prevented me from asserting causal relations. Second, the DHS did not include measures of gender ideology, which likely influences the prevalence of IPV against women (Yount & Li, 2009). I controlled for several potential confounders, including religious denomination, which is likely to promote views regarding gender roles and geographical location, which may capture differences in gender ideology. Third, as less than 0.2% of women indicated that they initiated physical violence toward their spouse, I could not include this variable as a control. In the future, longitudinal analyses are needed to examine how changes in the economic status of individual women affect their IPV risk over time. The relationship between women’s employment and physical IPV also merits further exploration to pinpoint whether it is the empowerment or necessity of employment that offers protection against IPV. Finally, as this study relied on women’s self-reports of all variables included in the analysis, future work should consider incorporating dyadic data.

## Supplemental Material

sj-pdf-1-vaw-10.1177_1077801221992877 – Supplemental material for Gender, Resources, and Intimate Partner Violence Against Women in Egypt Before and After the Arab Spring Click here for additional data file. Supplemental material, sj-pdf-1-vaw-10.1177_1077801221992877 for Gender, Resources, and Intimate Partner Violence Against Women in Egypt Before and After the Arab Spring by Mariam Abouelenin in Violence Against Women
