# Supplementary material for: Gender, Resources, and Intimate Partner Violence Against Women in Egypt Before and After the Arab Spring
Source: Violence Against Women. 2021 Mar 3;28(2):347–74. doi: 10.1177/1077801221992877 (PMC8721615; doi:10.1177/1077801221992877)
Supplement: sj-pdf-1-vaw-10.1177_1077801221992877 – Supplemental material for Gender, Resources, and Intimate Partner Violence Against Women in Egypt Before and After the Arab Spring [file sj-pdf-1-vaw-10.1177_1077801221992877.pdf]

GENDER, RESOURCES, AND INTIMATE PARTNER VIOLENCE AGAINST WOMEN IN EGYPT BEFORE AND AFTER THE ARAB SPRING.

APPENDIX A

Legal Response to Intimate Partner Violence Against Women in Egypt

In Egypt, intimate partner violence (IPV) against women is still a cultural taboo. Over the past decade, the abuse and oppression of women has gradually been identified as a cause for social concern by nongovernmental organisations and government officials. This has resulted in some changes to legislation focused on expanding women's rights, such as the ability to obtain a no-fault divorce (*khul*), collect court-ordered alimony, and extend legal custody of children until the age of 15 (Al-Sharmani, 2010). By contrast, while violence resulting in outwardly visible injuries is considered a felony under Egyptian law, IPV against women is not criminalised (Ammar, 2006). In the absence of visible bruising or skin trauma, the impact of IPV on women is regarded as not 'severe enough' to warrant intervention. This legal interpretation is further complicated by socioeconomic status. Rural poor women must evidence more severe harm compared with their richer urban counterparts (Ammar, 2006). This is based on the judicial assumption that the poor are accustomed to harsher treatment and, therefore, can endure more extreme forms of maltreatment.

The preservation of marriage and family relations still dominates in the assessment of court cases involving IPV against women. Cultural norms that favour family privacy and cohesion over women's well-being have meant that judges regularly suspend legal proceedings to encourage spousal reconciliation (Ammar, 2006). In addition, reluctances in addressing IPV against women are often defended on the grounds of Shari'a (Islamic Law) regarding the permissibility of violence. Specifically, Article 60 of the Egyptian Penal Law states that "the provisions of the Penal Code shall not apply to any deed committed in good faith[1], pursuant to a right determined by virtue of the Shari'a". That is, the use of IPV against

women who fail to uphold their religious duty[2] is seen as just and warranted. Thus, the difficulty of receiving legal support means that female victims of IPV are unlikely to report the violence, especially in the case of psychological IPV where injuries may be less observable.

APPENDIX B

Empirical Model

As explained in the main body of the paper, a two-stage least squares (2SLS) estimation method is applied to address issues of omitted variable bias and bidirectional correlation. The 2SLS model consists of two linear regression estimates as specified in equations (1) and (2). The first-stage regression is defined by:

$$WEM = \beta 0 + \beta 1 Res + \beta 2 Gov + \beta 3 COV + u \tag{1}$$

where the endogenous variable, women's employment (WEM), is predicted by the instruments, the number of residents (Res), the governorate average of women's employment (Gov) and exogenous variables (COV). In the second-stage equation, women's intimate partner violence risk (IPV) is regressed on the predicted values of women's employment ($\widehat{WEM}$) from the first stage and all other exogenous variables (COV):

$$IPV = \beta 0 + \beta 1 \widehat{WEM} + \beta 2 COV + u \tag{2}$$

APPENDIX C

Instrumental Variable

The 2005 and 2014 Egypt Demographic and Health Survey (EDHS) collected data on the number of usual residents and visitors in a household. All usual residents and visitors are

listed on the household schedule. The EDHS define a household as a group of individuals who live, sleep, and eat together, irrespective of whether they are related or not (ICF International, 2020). A usual resident of a household is any individual who normally lives in the household. A visitor is someone who slept in the household the night before the interview but lives somewhere else. For example, a domestic worker who normally lives and sleeps in the household would be recorded as a usual resident and listed in the household schedule. Similarly, an individual who slept in the household the night before but does not normally live there would be recorded as a visitor and listed in the household schedule. By contrast, an individual who does not usually live in the household and did not sleep there the previous night would be omitted from the household schedule. The usual residents of the household can be identified using the resident variable, which is also available in the EDHS.

There are a few additional reasons why the number of usual residents is a plausible instrumental variable for women's employment. Previous research has identified a link between living arrangements and constraints to women's labour market participation (Shen et al., 2016). In Arab countries such as Egypt, where traditional patriarchal values and practices are widespread, household units consisting of in-laws, parents, children, aunts, uncles, and cousins remain common. The availability of other household members may function as either a source of support or an added care burden. If members help with chores and childcare, then the time women devote to the household can decrease (Shen et al., 2016). This may help women overcome domestic work barriers to paid employment[3]. Unlike Western societies, where nursing homes are a viable care option, in Egypt, long-term care facilities are scarce and viewed as culturally unacceptable (Yount, 2005b). This means that the social obligation for elderly care falls entirely on families (Yount, 2005b). As the number of household members in need of care increases, this may place greater demands on women and reduce their ability to engage in employment.

APPENDIX D

<u>More Details About Terms that Appear in the Text</u>

The main text includes subject and country-specific terminology. In this Appendix, I follow the order in which the terms appear in the main text and provide more details about their meaning.

D1. Introduction

"I explore these relationships in the context of the events that took place during the **Arab Spring**" refers to a series of protests that took place in the Arab world between 2010-2011.

D2. Social and Economic Changes in Egypt Before and After the Arab Spring

"The prevalence of physical and psychological violence in Egypt among *ever-married women* is reported" refers to women who were married at least once before, regardless of their current marital status.

D3. Data and Sample

"The sample was stratified by six main regions: urban *governorates*, urban Lower Egypt, rural Lower Egypt, urban Upper Egypt, rural Upper Egypt and the Frontier *governorates*" refers to an administrative division. Egypt has 27 governorates: Cairo, Alexandria, Port Said, Suez, Damietta, Dakahlia, Sharqia, Al Qalyubia, Kafr El-Sheikh, Gharbia, Menoufia, El Beheira, Ismailia, Giza, Beni Suef, Faiyum, Minya, Assiut, Sohag, Qena, Luxor, Aswan, Matrouh, New Valley, North Sinai, South Sinai, and Red Sea.

APPENDIX E

First-Stage Estimates with Both Instruments

Table E1.
First-stage estimates. Instruments: residents and governorate average (N = 11,319)

	Women's employment<br>B<br>(SE)
Residents	–.01*<br>(.00)
Governorate average	1.14***<br>(.25)
White-collar worker (ref: blue collar & unemployed)	.02***<br>(.01)
Relative education (ref: H = W)	
W > H	–.03***<br>(.01)
H > W	–.01<br>(.01)
Educational distance	–.01<br>(.01)
Women's education (ref: none)	
Primary	.01<br>(.02)
Secondary	.03*<br>(.01)
Higher	.27***<br>(.02)
All other covariates	✓
Intercept	–.35***<br>(.05)

Note. H = W = both spouses educated to the same level. W > H = wife is better educated. H > W = husband is better educated. Weighted statistics with unweighted sample size.
† $p < .10$; * $p < .05$; ** $p < .01$; *** $p < .001$ (two-tailed tests)

APPENDIX F

Interactions

Figure F1.

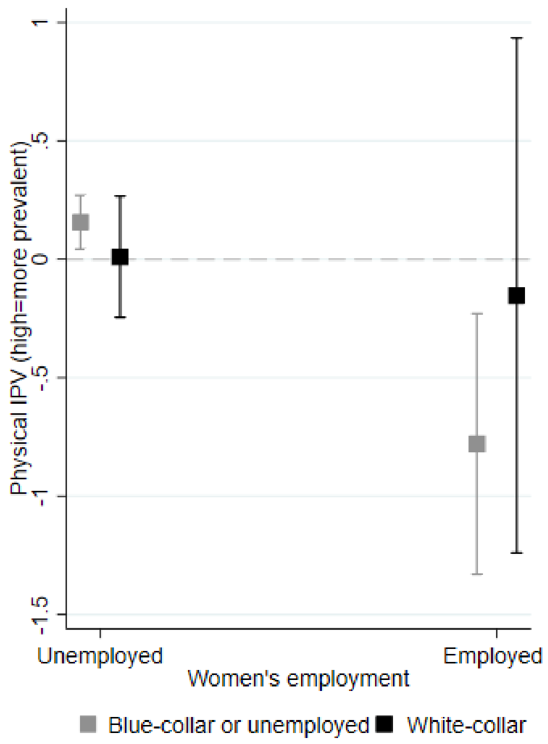

Note. N = 11,319 women. Predictive margins of the interaction effect between spouses' employment status and women's employment status.

NOTES

1. Violence committed in good faith is described as not severe, not directed at the face and not aimed at fatal blow areas (Ammar, 2006).
2. Including disobeying the spouse.
3. Another common strategy is enlisting the help of domestic workers who reside in the home on a full or part-time basis. To account for this, I also check sensitivity of results to include household visitors. The direction, size, and significance of the key predictors were consistent with the results presented in the paper.
